# Supplementary material for: Programmed multimaterial assembly by synergized 3D printing and freeform laser induction
Source: Nat Commun. 2024 May 28;15:4541. doi: 10.1038/s41467-024-48919-5 (PMC11133382; doi:10.1038/s41467-024-48919-5)
Supplement: Supplementary file 1 — Supplementary Information [file 41467_2024_48919_MOESM1_ESM.pdf]

Supplementary Information for

Programmed Multimaterial Assembly by Synergized 3D Printing and Freeform Laser Induction

Bujingda Zheng[1], Yunchao Xie[1], Shichen Xu[2], Andrew Meng[3], Shaoyun Wang[1], Yuchao Wu[1], Shuhong Yang[4], Caixia Wan[4], Guoliang Huang[1], James M Tour[2,5,6], and Jian Lin[1*]

[1]Department of Mechanical and Aerospace Engineering

University of Missouri, Columbia, MO 65201, United States

[2]Department of Chemistry

Rice University, 6100 Main Street, Houston, 77005, Texas, USA

[3]Department of Physics and Astronomy

University of Missouri, Columbia, MO 65201, United States

[4]Department of Chemical and Biomedical Engineering

University of Missouri, Columbia, MO 65201, United States

[5]Department of Materials Science and Nano Engineering

Rice University, 6100 Main Street, Houston, 77005, Texas, USA

[6]Smalley Institute for Nanoscale Science and Technology

Rice University, 6100 Main Street, Houston, 77005, Texas, USA

*Email: linjian@missouri.edu

Supplementary Figures

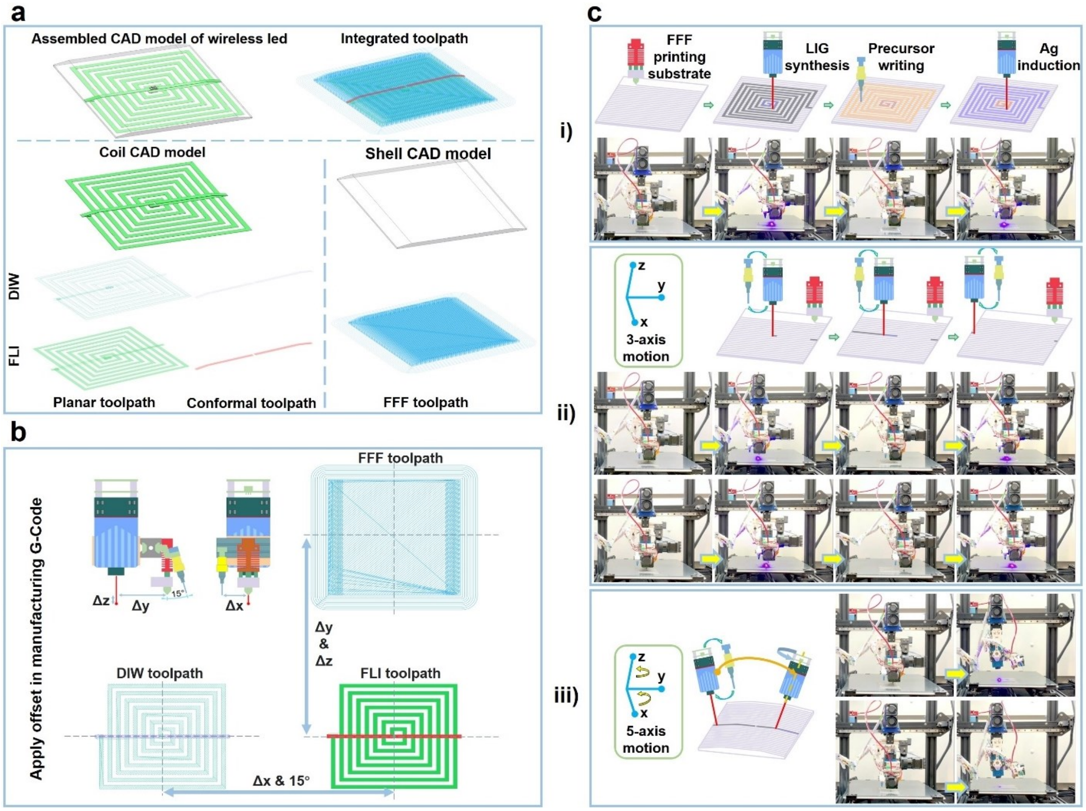

Supplementary Figure 1| Workflow of fabricating a wireless LED using FMAP. (**a**) Design and modeling of electrodes for a wireless LED and respective G-code generation. The coil and shell electrodes are modeled independently. Subsequently, the toolpaths for both the coil and shell are generated using a slicer tool, which are tailored to the specific requirements of FLI, DIW, and FFF. (**b**) Toolpaths for FFF, FLI, and DIW. To achieve precise positioning of the three end effectors, the toolpaths are integrated with their respective offset parameters. (**c**) Time-lapse images of the different fabrication steps for a wireless LED: (**i**) printing of a polycarbonate (PC) substrate; (**ii**) multilayer LIG/Ag electrodes are fabricated within the printed PC structure; (**iii**) conformal patterning of the LIG/Ag electrodes on the surface of the PC structure. Throughout this procedure, all actuators work synergistically to ensure that the laser beam remains perpendicular to the target 3D surface.

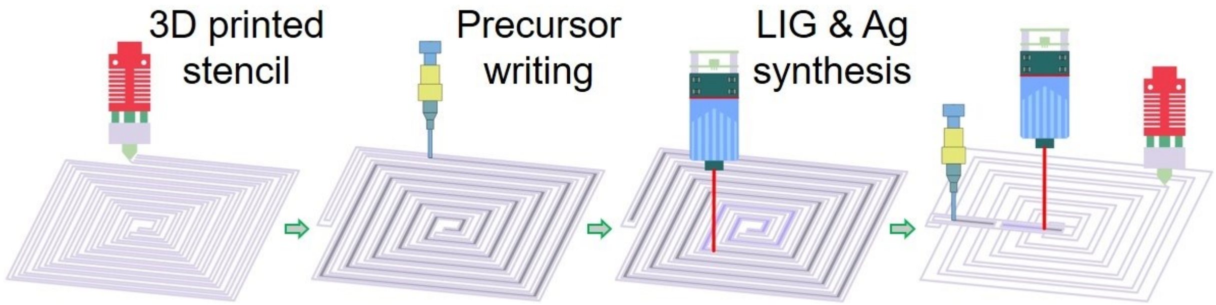

Supplementary Figure 2| Workflow of using FMAP to fabricate a 3D wireless LED with LIG/Ag electrodes made from TPU and a precursor mixture, which consists of lignin and silver citrate. Note: the reason of including lignin in the silver citrate ink is that it can help laser induction for LIG formation from polymers such as TPU and PETG which are not carbonized by laser directly.

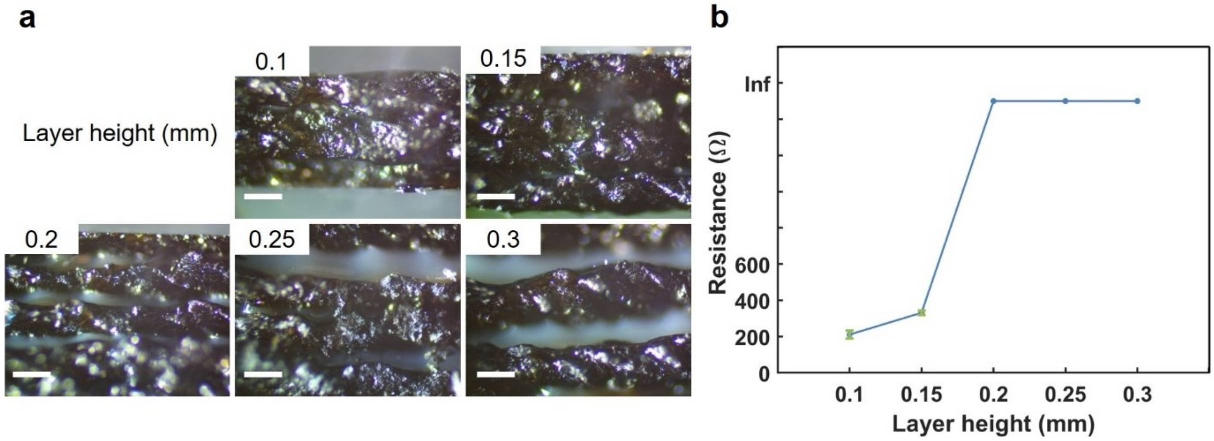

Supplementary Figure 3| Characterization on the LIG induced from PC printed with different layer heights. (**a**) Cross-sectional optical images of the LIG induced from PC printed with five different layer heights, a scan rate of 300mm/min and power of 2.5 W. Scale bar: 200 μm. (**b**) Change of the resistance of LIG in the z-axis direction vs. the layer height. Error bars indicate the standard deviation obtained from more than 5 sheet resistance measurements. Note: because of decreased conductivity along the z-axis as the increased layer height, a layer height of 0.15 is typically employed for all the showcased devices in this study.

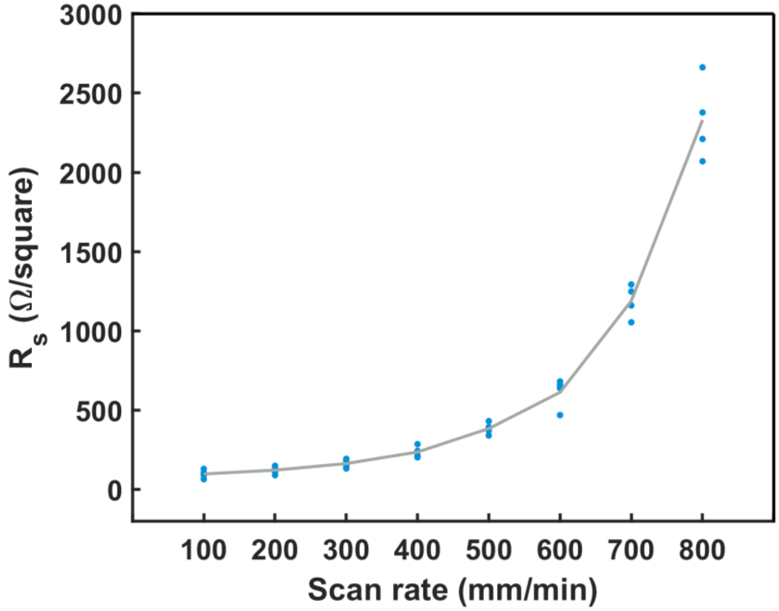

Supplementary Figure 4| sheet resistance of LIG as a function of laser scan rate.

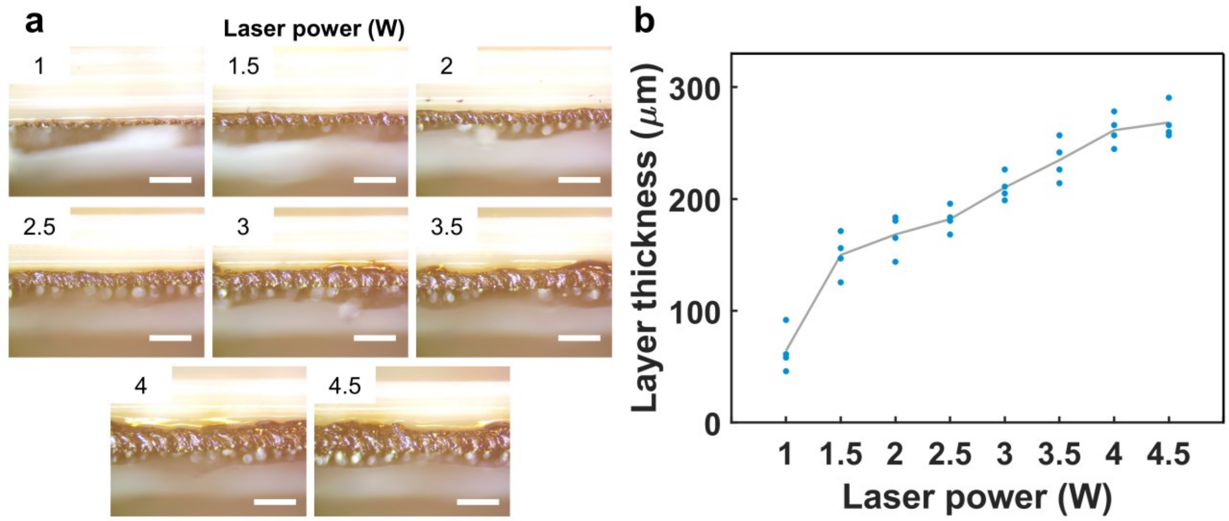

Supplementary Figure S5| Relationship between laser power and the LIG thickness. (**a**) Photographs showing cross sections of 8 LIG samples made by different laser powers. (**b**) LIG thickness as a function of laser power. Scale bar: 500 μm.

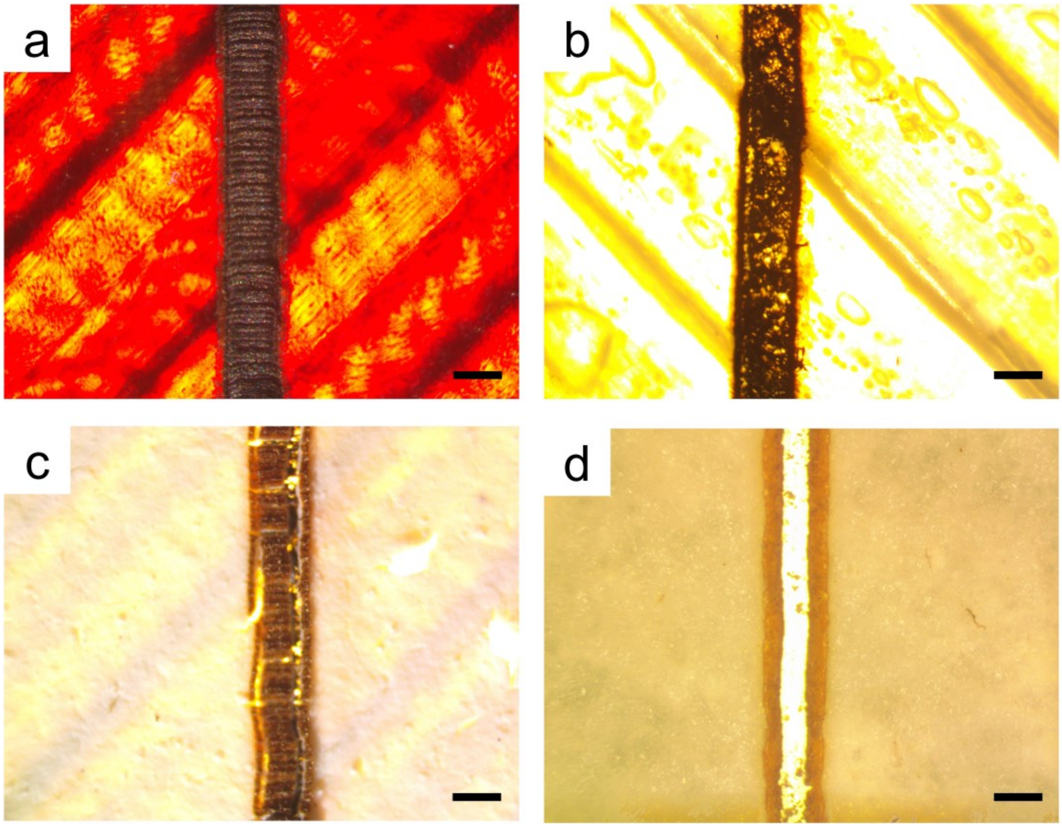

Supplementary Figure 6| Resolution of laser induction on various substrates. (**a**) A photograph showing a LIG strip with 218 μm linewidth induced from lignin on PETG substrate, (**b**) A photograph showing a LIG strip with 235 μm linewidth induced from PVDF substrate, (**c**) A photograph showing a LIG and Ag strip with 247 μm linewidth induced from PC and Ag precursor, (**d**) A photograph showing a 104 μm-width laser induced Ag on PC. Scale bar: 200 μm.

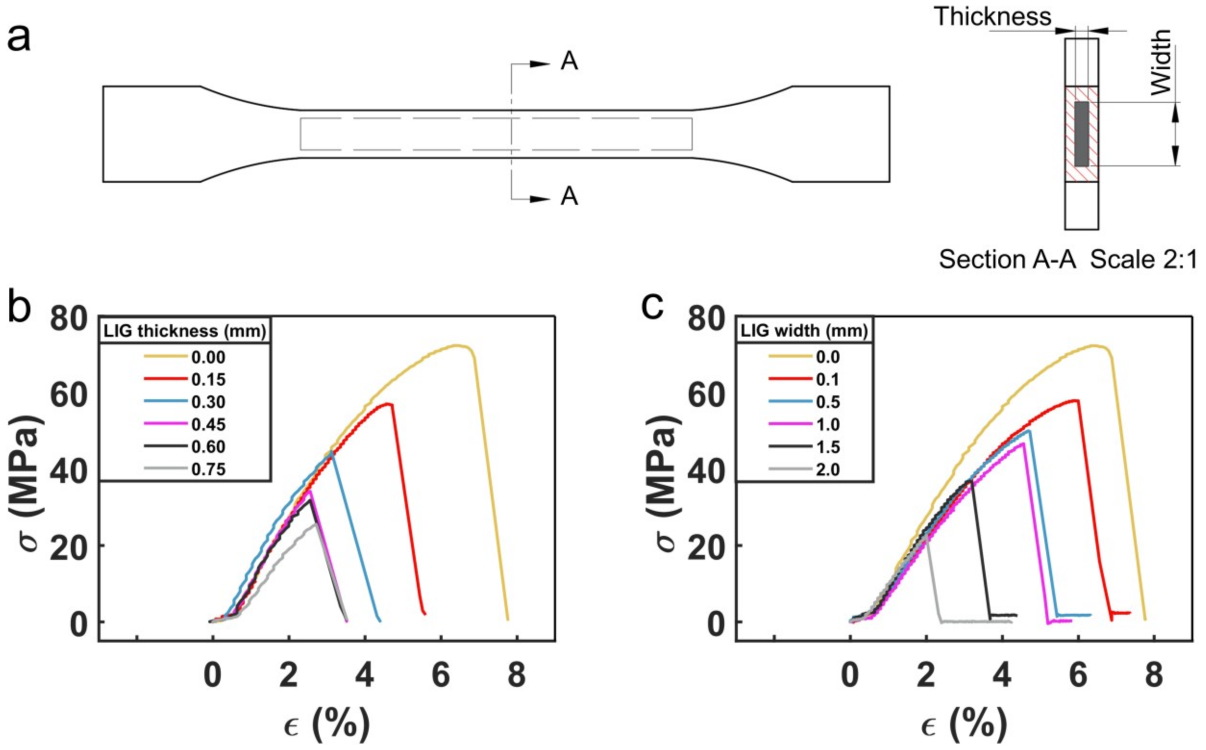

Supplementary Figure 7| Tensile testing was conducted on PC samples featuring different dimensions of embedded LIG. (**a**) A schematic illustrates the structure of a tensile testing specimen with LIG embeded inside. (**b**) Stress-strain curves of PC specimens with varied thicknessed LIG. (**c**) Stress-strain curves of PC specimens with varied widthed LIG.

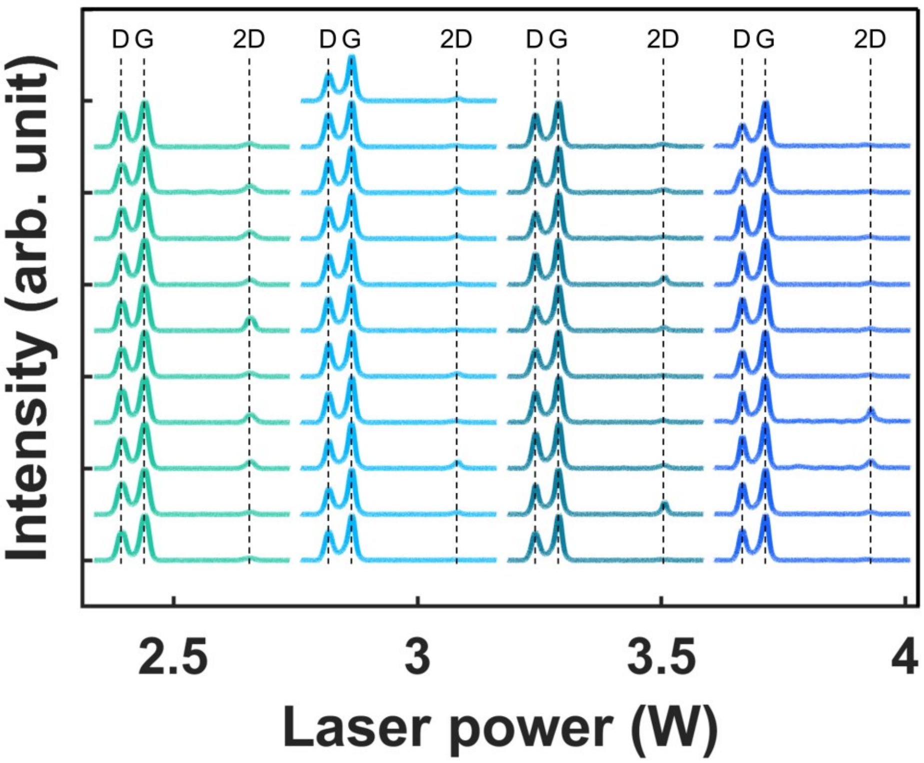

Supplementary Figure 8| Raman spectra of LIG from PC induced with different laser powers.

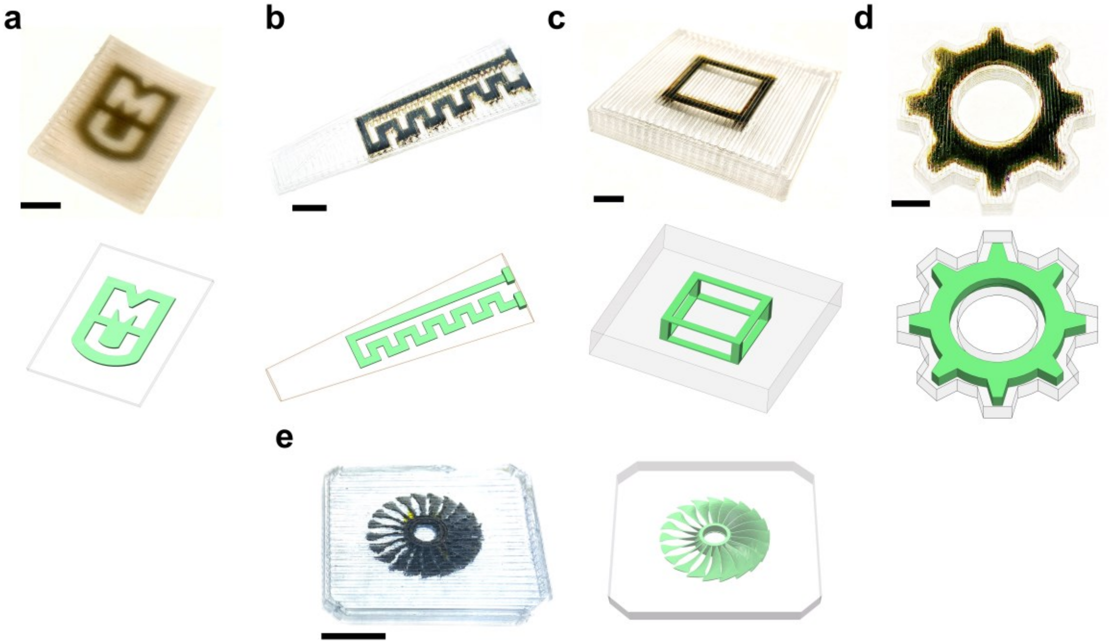

Supplementary Figure 9| Various 3D structures with imbedded LIG patterns fabricated by FMAP. (**a**) A MU logo made from LIG embedded in a printed PVDF structure. (**b**) An airfoil embedded with a LIG Zigzag pattern in PC. (**c**) A LIG lattice structure embedded in a PC cuboid. (**d**) A printed PC gear embedded with LIG. (**e**) A 3D LIG fan embedded in PC. Scale bar: 10 mm.

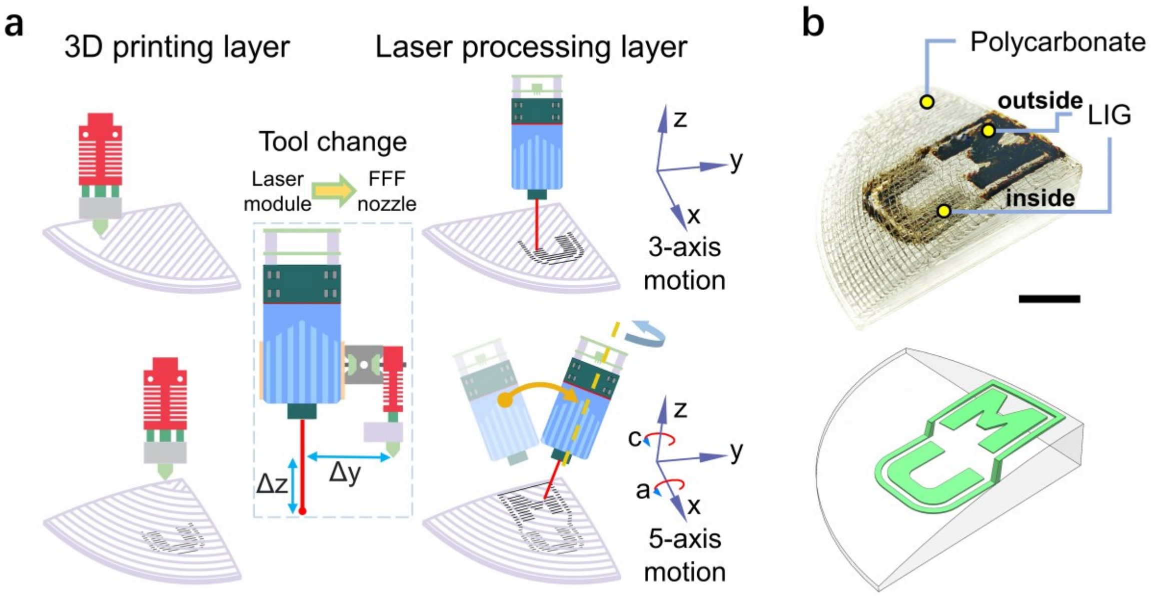

Supplementary Figure 10| A spatially patterned LIG 'MU' logo fabricated by FMAP. (**a**) Workflow of fabricating the 3D 'MU' logo. (**b**) A scheme and a photograph showing the structure of the fabricated 3D 'MU' logo. Scale bar: 10 mm.

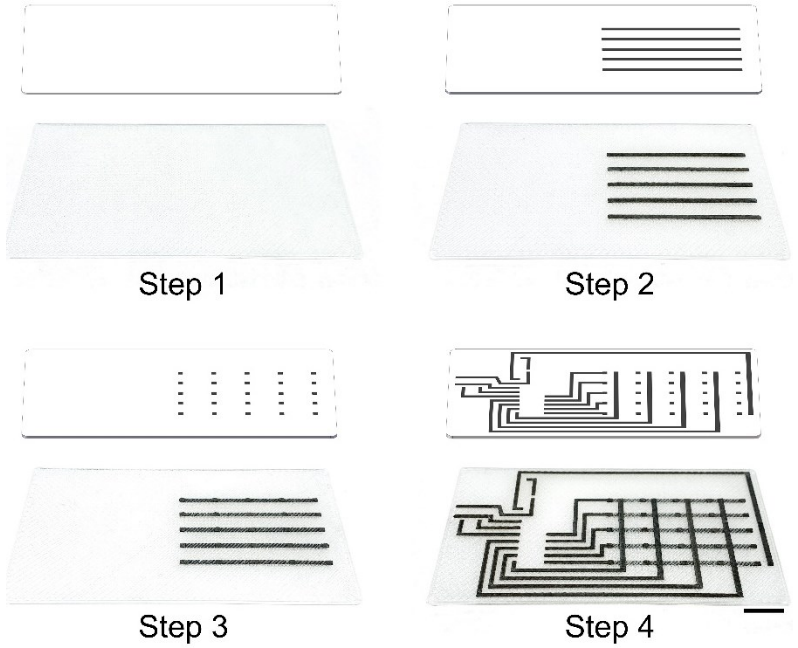

Supplementary Figure 11| Photographs showing fabrication steps for the crossbar LED array by FMAP. Scale bar: 10 mm.

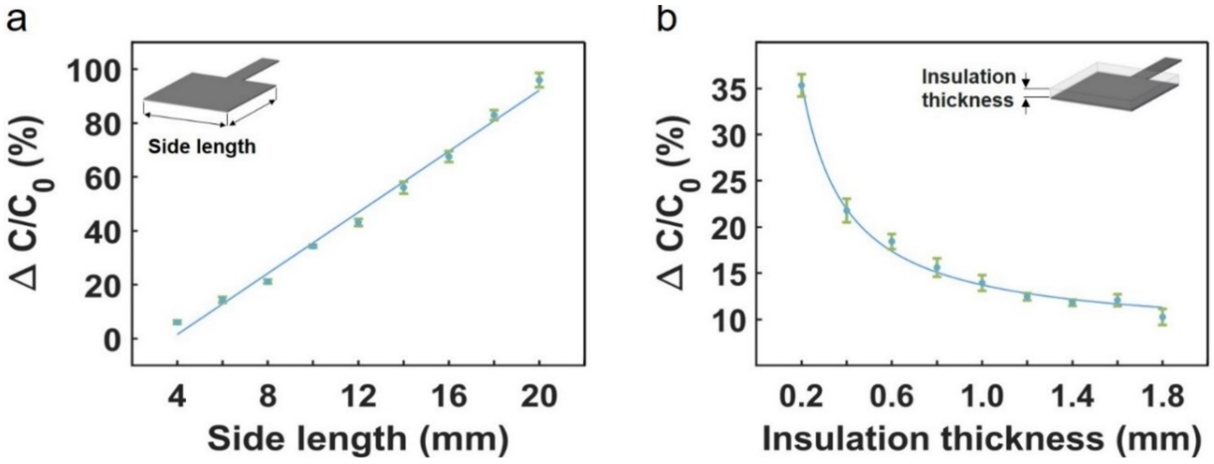

Supplementary Figure 12| Capacitance changes as a function of (a) length of electrodes and (b) thickness of the insulator in between the two electrodes. The data indicate trends consistent with the capacitance calculation, where capacitance is positively proportional to the electrode length while inversely proportional to the distance between electrodes. Repeated measurements were taken from the same samples For standard deviation calculation.

The experimental outcomes well agree with the capacitance formula, $C = \frac{\epsilon_0 A}{d}$, where A represents the electrode overlap area and d signifies the distance between the electrodes. As illustrated in Figure S8, it is evident that the variation in capacitance is directly proportional to the electrode area and inversely proportional to the electrode separation distance.

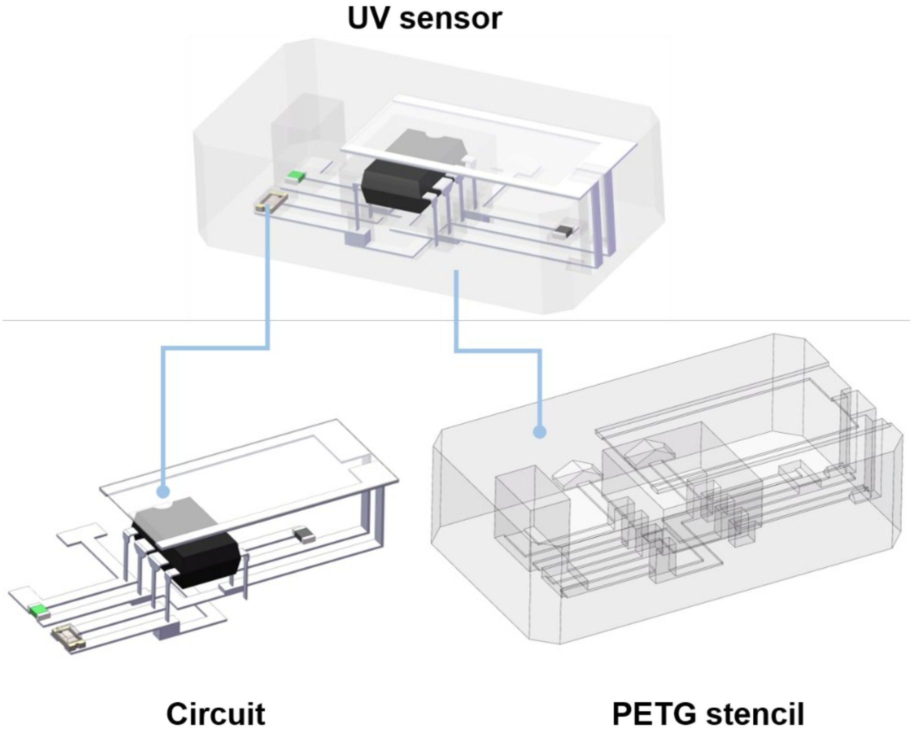

Supplementary Figure 13| Schematic showing the circuits and electrical components for the UV sensor. The PETG stencil for the Ag electrodes is also illustrated.

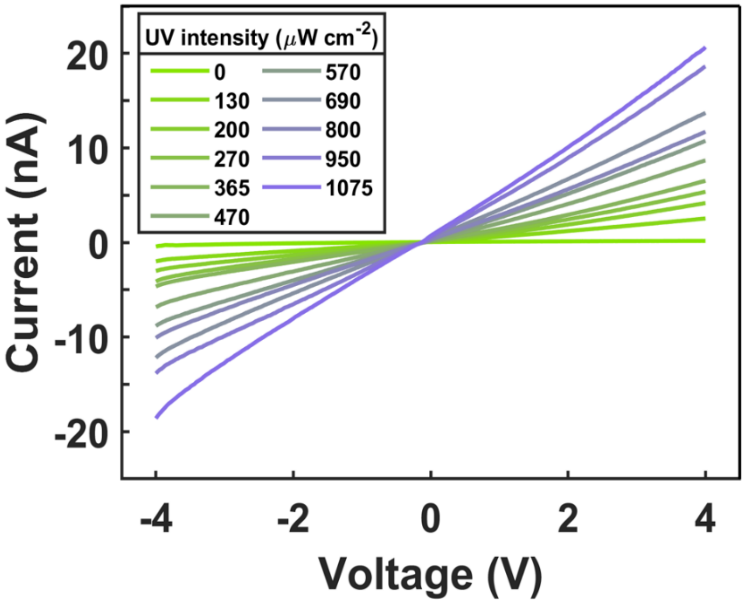

Supplementary Figure 14| I-V curves of the UV sensor at varying UV light intensities ranging from 0 to 1075 μW/cm^2.

Among these I-V (current-voltage) curves, the linear behavior can be ascribed to the Ohmic connection between the ZnO material and the Ag electrode. To illustrate, at an incident power (P) of 130 μW/cm^2, the I-V curve exhibits a slope of 5.38e-10. As the incident power increase, the slope proportionally increases, reaching 4.67e-9 at P = 1075 μW/cm^2. This experimental observation can be attributed to the diminishing resistance from the creation of charge carriers induced by UV.

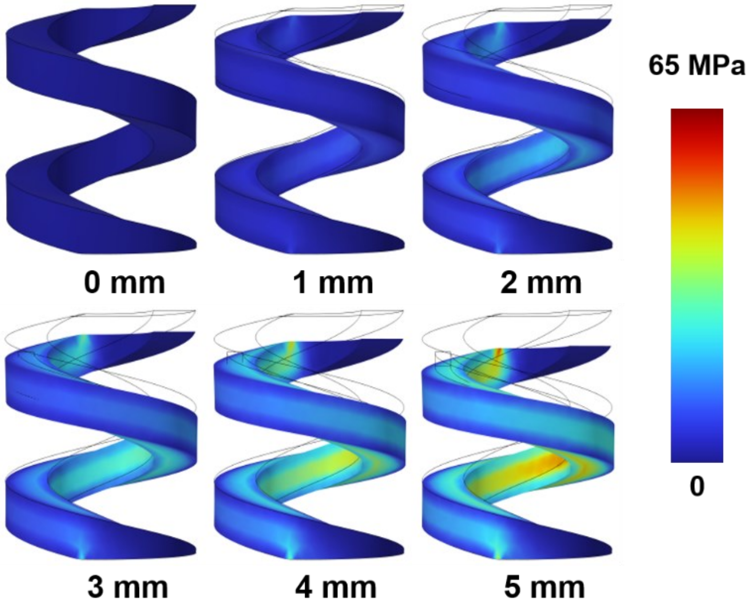

Supplementary Figure 15| Von Mises stress distribution of the spring under displacements of 0-5 mm derived from FEA simulation.

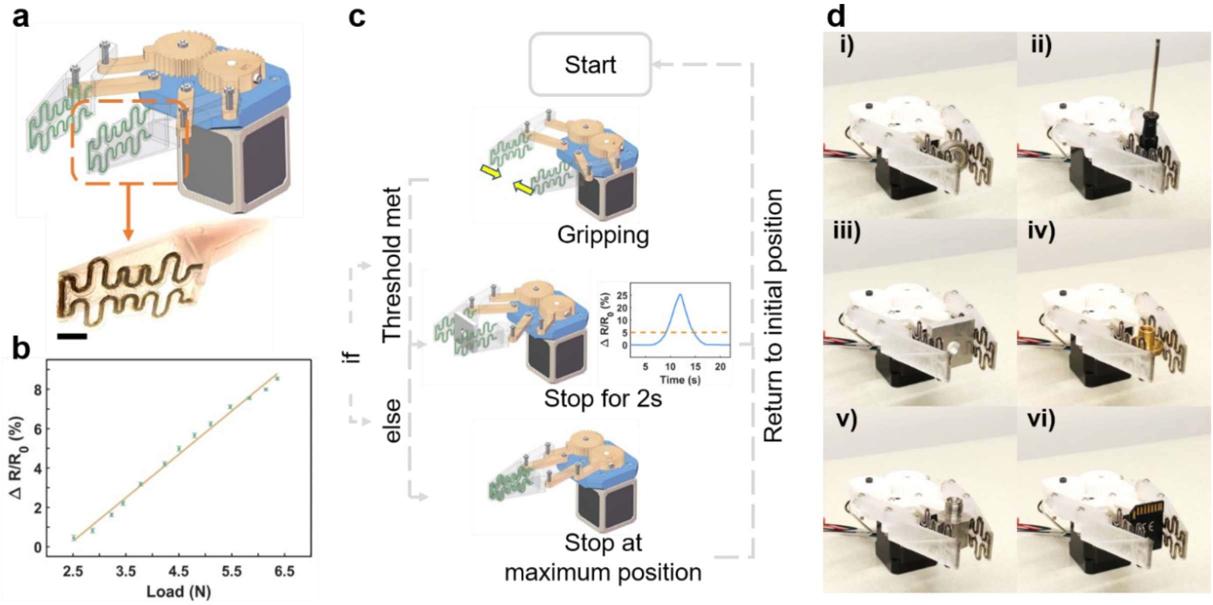

Supplementary Figure 16 Performance of a fabricated gripper with an embedded LIG strain sensor. (**a**) A schematic showing the assembled manipulator and a photograph showing a strain sensor embedded gripper fabricated by FMAP. (**b**) Resistance change as a function of load. (**c**) Scheme showing the workflow of the feedback loop control. (**d**) Photographs showing the manipulator adeptly manipulating: **(i)** a ball bearing; **(ii)** a screwdriver; **(iii)** an aluminum block; **(iv)** a t-nut; **(v)** a brass adapter; and **(vi)** a SD card. Scale bar: 10 mm.

Note: In response to varying gripping forces while manipulating objects, the LIG strain sensor undergoes corresponding deformations, generating distinct electrical signals. This behavior is illustrated in **Fig. S16b**, which displays the linear response of the strain sensor as the applied load changes within the range of 2.5N to 6.5N. Accordingly, a force feedback control algorithm was developed. It halts the gripper once the electrical strain attains a threshold of 5%, as shown in **Fig. S16c**. Implementing this control scheme empowers the gripper to adeptly grip a range of objects, including bearings, screwdrivers, aluminum blocks, T-nuts, brass adapters, and SD cards (**Fig. S16d**).

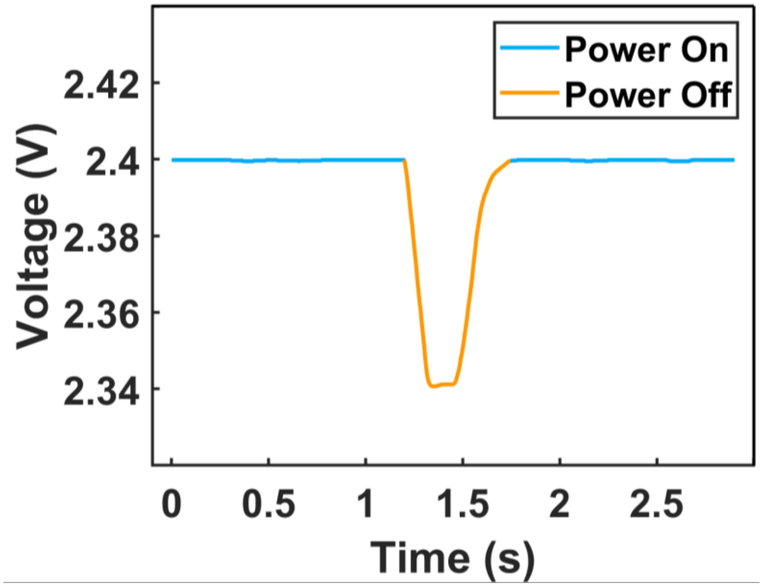

Supplementary Figure 17| Voltage response of a Hall sensor as an approached micro electromagnet is switched on and off.
