# Supplementary material for: Programmed multimaterial assembly by synergized 3D printing and freeform laser induction
Source: Nat Commun. 2024 May 28;15:4541. doi: 10.1038/s41467-024-48919-5 (PMC11133382; doi:10.1038/s41467-024-48919-5)
Supplement: Supplementary file 3 — Description of Additional Supplementary Files [file 41467_2024_48919_MOESM3_ESM.pdf]

Description of additional supplementary files

Supplementary movies
File name: Supplementary movie 1
Description: 'The FMAP fabrication process of a wireless LED'

File name: Supplementary movie 2
Description: 'Testing the FMAP-fabricated wireless LED'

File name: Supplementary movie 3
Description: 'Testing the FMAP-fabricated wireless LED on flexible substrate'

File name: Supplementary movie 4
Description: 'The freeform FMAP fabrication process of a 3D 'MU' logo'

File name: Supplementary movie 5
Description: 'Testing the FMAP-fabricated crossbar LED array'

File name: Supplementary movie 6
Description: 'Using the FMAP-fabricated touchpad for wireless LED array control'

File name: Supplementary movie 7
Description: 'Using the FMAP-fabricated slider for LED brightness control'

File name: Supplementary movie 8
Description: 'Testing the FMAP-fabricated UV sensor'

File name: Supplementary movie 9
Description: 'Electronic response of the FMAP-fabricated LIG strain sensor-embedded spring'

File name: Supplementary movie 10
Description: 'Haptic grasping with the FAMP-fabricated robotic manipulator embedded with LIG strain sensors'

File name: Supplementary movie 11
Description: 'Evaluating the FMAP-fabricated electromagnet for rotational encoder'

File name: Supplementary movie 12
Description: 'FEA simulation results for microfluidic channel under different flow rates'
